# Neutron and photon science facilities at a crossroads: embracing innovation in a changing world

**DOI:** 10.1107/S2052252526004161

**Published:** 2026-04-30

**Authors:** D. N. Argyriou

**Affiliations:** a Advanced Light Source 1 Cyclotron Road Lawrence Berkeley National Laboratory Berkeley CA 94720-8229 USA

**Keywords:** scientific user facilities, science funding, neutron science, photon science, light sources, artificial intelligence (AI), editorial

## Abstract

Sustaining public investment in neutron and photon science facilities demands breakthrough results comparable to a Higgs moment. Harnessing AI across their accumulated experimental data and collaborative programmes is what makes that possible.

Large-scale neutron and photon science facilities have been, and continue to be, indispensable tools for scientific discovery. Their contributions to Nobel-Prize-winning work, to advances in condensed matter physics, structural biology, materials science and energy research are beyond dispute. Yet scientific impact alone is no longer a sufficient argument for sustaining the public investment these facilities require. The question that governments, funding agencies and treasury ministries are now asking – with increasing bluntness – is a straightforward one: with the winds of economic, energy and technological change, how will these facilities remain relevant, and will there be a return on the investment to build and operate them? It is a question the community has not yet answered convincingly enough.

The pressures are real on both sides of the Atlantic. In the United States, a shift in how Washington views basic research is highlighted by the tech CEO majority presence in the President’s Council of Advisors on Science and Technology. In the United Kingdom, the Science and Technology Facilities Council – which funds Diamond Light Source and ISIS – faces the most drastic budget rebalancing in its history, with physics and astronomy grants set to fall to around 70% of their 2024–2025 levels. In Germany, the Fraunhofer-Gesellschaft and the expert commission on research and innovation have both called for faster translation of research into economic value – the same pressure, in a different register. Defence spending is rising. Governments are re-prioritizing. And the geopolitical dimension adds urgency: Asia is the only region where neutron and photon science capacity is set to increase substantially over the next two decades. China’s High Energy Photon Source has entered operations as a fourth-generation synchrotron directly competitive with the upgraded Advanced Photon Source and the ESRF; more Chinese X-ray sources are under construction, and more are in advanced planning across south-east Asia. The China Spallation Neutron Source is expanding. The countries that lead in this infrastructure will carry real advantages in semiconductor manufacturing, battery chemistry, pharmaceutical development and critical materials for defence.

The central challenge for photon and neutron sources, then, is to demonstrate value in terms that governments and funding agencies can recognize and act on. There is a narrow window to do this, and artificial intelligence has opened it – not only as a tool for data analysis, but as a bridge to industry collaboration and a mechanism for delivering the kind of high-visibility results that make the case for sustained investment. Seizing that opportunity will require a change in how these facilities think about their scientific mission, how they measure their own success, and how willing they are to innovate in areas where progress has, frankly, been too slow.

## 1. A unique and underappreciated challenge

To understand what is at stake, it is worth being clear about what kind of scientific infrastructure neutron and photon facilities actually are – because they are fundamentally different from the other great classes of large-scale science, and that difference defines both their value and their communications problem.

Large telescopes and particle colliders probe nature directly. The telescope looks outward to understand the structure and evolution of the universe; the collider looks inward, to the fundamental constituents of matter. In both cases, the facility and the scientific objective are inseparable. The community coalesces around a singular grand question, and when results come they carry unmistakable force. The discovery of the Higgs boson involved close to 3000 authors (Aad, 2012 ▸; Chatrchyan, 2012 ▸), the observation of gravitational waves just over a thousand (Abbott, 2016 ▸) – results reported globally, communicating their significance instantly. These are facilities that produce Higgs moments.

Neutron and photon facilities are different in a fundamental way. Here, the object being investigated is not nature at large – out in the stars or at the scale of fundamental particles – but nature inside a tiny sample made in a laboratory. The science is in the sample. A principal investigator and their team create or prepare that sample – a quantum material exhibiting novel magnetic behaviour, a protein crystal from a candidate cancer therapeutic, a battery electrode after thousands of charge cycles – and bring it to the facility for measurement. The facility is, in essence, a universal microscope through which an enormously diverse population of researchers – condensed matter physicists, structural biologists, materials engineers, chemists, geologists, archaeologists – pass their entirely different questions. There is no singular unifying scientific objective. There is instead a continuous, distributed accumulation of knowledge across many disciplines simultaneously, each contribution significant within its field, but none individually carrying the scientific or technological weight of a single transformative discovery. This breadth is genuinely one of the greatest strengths of these facilities. It is also their most serious communications problem: the impact of their scientific productivity is diluted across many papers, even though tens of thousands of researchers use them every year.

One dimension of value that is consistently underweighted in public debate is the role these facilities play in training scientists and engineers, and in building the expertise of the small- and medium-sized enterprises that support their construction and operations – precision engineering companies, cryogenics specialists, detector manufacturers, control systems developers. This industrial supply chain represents a form of national technological capacity and capability that cannot be rapidly recreated once it is lost. It is a return on investment that rarely appears in any publication count, but it is nevertheless real, substantial and massively impactful.

## 2. What is already possible: two proof points

The case that fourth-generation light source performance combined with AI and high-performance computing (HPC) can deliver genuinely transformative science is not theoretical. At the ESRF, the Extremely Brilliant Source (EBS) enabled the development of hierarchical phase-contrast tomography (HiP-CT) (Walsh *et al.*, 2021 ▸): a technique that images intact human organs non-destructively, from whole-organ scale at 20 µm resolution down to individual cellular structures at less than one micron. The Human Organ Atlas (Walsh *et al.*, 2026 ▸) – an open-access repository of three-dimensional images of more than 50 human organs, created with nine institutions worldwide – has already revealed previously unseen vascular damage in COVID-19 lungs, provided new structural insights into cardiac disorders, and opened avenues in gynaecological pathology and connectomics (Azevedo *et al.*, 2024 ▸). None of this was possible without combining EBS beam performance with high-performance computing capable of handling the data volumes involved. Source upgrade plus AI plus computing – that combination is what delivers results at this scale.

The relationship between synchrotron science and microelectronics runs deeper than inspection. It extends to the very technique used to manufacture today’s most advanced chips. Since the 1990s, Berkeley Lab’s Center for X-Ray Optics harnessed the vacuum ultraviolet light of the Advanced Light Source to pioneer extreme ultraviolet (EUV) lithography – the process now used by ASML and the global semiconductor industry to print features at 13.5 nm wavelength in the current production of every leading-edge microprocessor and memory chip. That technology transfer, from a synchrotron beamline to a USD 27 billion market, is one of the most consequential industrial contributions any light source has made. It is also a model for what is possible when facilities engage seriously with industry around a shared technical challenge. Synchrotron science can now add a complementary capability: non-destructive imaging of the chips once made. Ptychographic X-ray lamino­graphy demonstrated three-dimensional imaging of 16 nm FinFET chips with 18.9 nm resolution, with burst ptychography subsequently achieving 4 nm resolution at 170 times faster acquisition at the Swiss Light Source (Holler *et al.*, 2019 ▸; Aidukas *et al.*, 2024 ▸). At the upgraded Advanced Photon Source, the first nano-lamino­graphy imaging of an integrated circuit on a US fourth-generation machine has now been demonstrated at 50 nm resolution (Nikitin *et al.*, 2025 ▸). Coherent imaging scales directly with source brilliance, and the APS-U’s advantage over the third-generation Swiss Light Source means that gap will close rapidly. The strategic relevance of this capability – for verifying that chips manufactured overseas conform to their design specifications – is self-evident in an era of semiconductor supply chain scrutiny.

## 3. The strategic opportunity: AI and the Higgs moment for the science of materials

These results illustrate both the opportunity and the urgency. The new generation of fourth-generation synchrotrons – the upgraded Advanced Photon Source, the ESRF-EBS, the forthcoming ALS upgrade – generate X-ray data at rates and volumes that traditional analysis workflows simply cannot keep pace with. A single experiment can produce petabytes of data in hours. If those data cannot be analysed at the rate they are generated, the scientific benefit of the source upgrade is not realized. AI-driven data reduction, autonomous experiment steering and machine-learning-enhanced structure solution are therefore not optional enhancements – they are the prerequisite for harvesting the scientific return on the billions invested in these facility upgrades.

**Figure 1:**
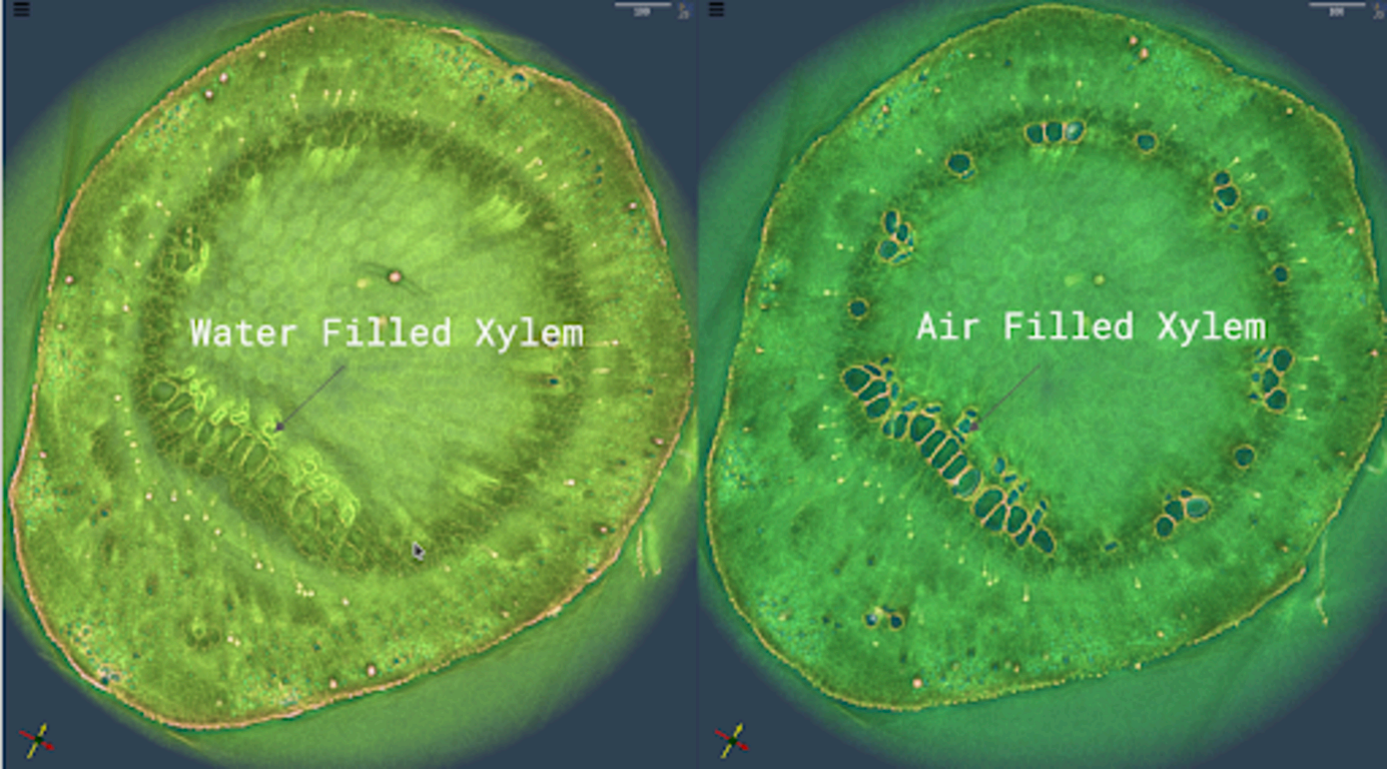
Water-filled versus air-filled xylem vessels revealed through AI-driven segmentation. By leveraging machine learning as part of the Genesis Mission and large-scale HPC inference, segmentation time is reduced from approximately one month of manual annotation to ∼8 minutes, enabling rapid, *in situ* analysis of plant hydraulic function during experiments. Plant sample provided by Andrew McElrone (UC Davies), data collected at beamline 8.3.2 at the Advanced Light Source, Berkeley Lab.

In the United States, this has been recognized at the highest level. The DOE’s Genesis Mission (The White House, 2025 ▸) – launched by Executive Order in November 2025 – is a national initiative aimed at using AI to double R&D productivity within a decade by linking supercomputers, experimental facilities and datasets into a single integrated platform. Central to this effort is SYNAPS-I: a multi-laboratory AI platform integrating foundation models across seven DOE user facilities (Lawrence Berkeley National Laboratory, 2026 ▸). Early versions of SYNAPS-I transform petabytes of imaging data into actionable knowledge in under 15 minutes – a task that previously took days – by automating the segmentation and characterization of materials at the beamline in real time. This is a genuine paradigm shift in how experiments are managed and how the next experimental steps are planned (see Fig. 1 ▸).

But the deeper opportunity is more significant still. Consider what these facilities collectively hold: decades of rich, multi-dimensional experimental data spanning condensed matter physics, structural biology, engineering, chemistry and beyond. Each individual experiment answered a narrow question. But the entire corpus – properly curated, standardized and subjected to AI analysis at scale – represents one of the most valuable untapped scientific knowledge bases likely in existence. By organizing a meaningful fraction of their scientific programme around large collaborative questions, and applying AI to synthesize results across many samples, conditions and research groups, these facilities could begin to deliver the kind of high-visibility, large-team scientific breakthroughs that governments and funding agencies recognize and fund. A Higgs moment for materials science or structural biology is not a fantasy. It is the logical destination of AI-enabled large-scale collaborative science, and these facilities are well placed to pursue it.

There is, however, a real danger alongside the opportunity. As facilities encode decades of accumulated instrument knowledge into AI-driven pipelines, that knowledge risks becoming inaccessible to the humans who will need to extend or correct those systems in the future. The generation of scientists who understood photon and neutron instruments at the level of their physics – who could extract the maximum from a difficult measurement through hard-won experimental intuition – is retiring. Their successors learn to use AI systems without necessarily acquiring the hard-won experience those systems encode. This is not an argument against the AI transition. It is an argument for designing training programmes that preserve human understanding alongside algorithmic capability, before that window closes.

## 4. What needs to change

The case for these facilities to governments needs to be made in three registers simultaneously – scientific, economic and strategic – and our community is currently strongest in the first and needs to work harder in the other two. The dominant metric of success – publications in high-impact journals – was a reasonable pr­oxy for scientific productivity in the era of individual investigator-driven experiments, but moving forward it may no longer be sufficient. Future metrics will need to encompass contributions to foundational scientific AI models trained on experimental data; participation in large collaborative research programmes with measurable strategic impact; and demonstrable influence on industrial innovation traceable to facility-enabled science. And they will need to encompass something harder to quantify but equally important: investment in training the next generation of scientists, engineers and AI practitioners who will operate, improve and ultimately re-imagine these facilities. The data accumulated at these facilities over decades are potentially one of the most valuable AI training datasets that we may know of for the next generation of materials science. Facilities that invest in making those data AI-ready, and that position themselves as foundational contributors to the scientific knowledge infrastructure of the coming decades, will be making an argument for their value that is genuinely durable.

Advanced technical performance alone has never been sufficient to justify the investment in large-scale facilities or to sustain their long-term operational costs. The true value of these facilities is realized over many decades by an engaged scientific community – but only if the facilities remain relevant, innovative and aligned with the world around them, and are not afraid to take some calculated risks along the way. Economic pressures and geopolitics may be closing the window for demonstrating that relevance faster than the community has yet appreciated. The facilities that seize it – that harness AI to unlock the latent value in their accumulated data, that build the large collaborative and industry-engaged programmes the AI opportunity demands, that train the next generation in science, AI and engineering, and that tell their story in the language that matters to those who fund them – will not merely survive the current pressures. They will emerge from them more indispensable than before.
